# Blockade of microglial adenosine A2A receptor impacts inflammatory mechanisms, reduces ARPE-19 cell dysfunction and prevents photoreceptor loss *in vitro*

**DOI:** 10.1038/s41598-018-20733-2

**Published:** 2018-02-02

**Authors:** M. H. Madeira, K. Rashid, A. F. Ambrósio, A. R. Santiago, T. Langmann

**Affiliations:** 1 Institute for Biomedical Imaging and Life Sciences (IBILI), Faculty of Medicine, University of Coimbra, Coimbra, Portugal; 2 CNC.IBILI Consortium, University of Coimbra, Coimbra, Portugal; 3 Laboratory for Experimental Immunology of the Eye, Department of Ophthalmology, University of Cologne, Cologne, Germany; 4 Association for Innovation and Biomedical Research on Light and Image (AIBILI), Coimbra, Portugal

## Abstract

Age-related macular degeneration (AMD) is characterized by pathological changes in the retinal pigment epithelium (RPE) and loss of photoreceptors. Growing evidence has demonstrated that reactive microglial cells trigger RPE dysfunction and loss of photoreceptors, and inflammasome pathways and complement activation contribute to AMD pathogenesis. We and others have previously shown that adenosine A_2A_ receptor (A_2A_R) blockade prevents microglia-mediated neuroinflammatory processes and mediates protection to the retina. However, it is still unknown whether blocking A_2A_R in microglia protects against the pathological features of AMD. Herein, we show that an A_2A_R antagonist, SCH58261, prevents the upregulation of the expression of pro-inflammatory mediators and the alterations in the complement system triggered by an inflammatory challenge in human microglial cells. Furthermore, blockade of A_2A_R in microglia decreases the inflammatory response, as well as complement and inflammasome activation, in ARPE-19 cells exposed to conditioned medium of activated microglia. Finally, we also show that blocking A_2A_R in human microglia increases the clearance of apoptotic photoreceptors. This study opens the possibility of using selective A_2A_R antagonists in therapy for AMD, by modulating the interplay between microglia, RPE and photoreceptors.

## Introduction

Age-related macular degeneration (AMD) is a major cause of vision loss worldwide and the leading cause of blindness in the elderly in developed countries^1^. The accumulation of cellular debris as drusen in the subretinal space, beneath the retinal pigment epithelium (RPE), is a main feature of early and intermediate AMD, which can progress to photoreceptor loss, representing the so-called dry AMD. Wet AMD is characterized by choroidal neovascularization, with new blood vessels arising from the choroid through the RPE layer into the outer retina, leading to photoreceptor dysfunction^2^.

Growing evidence supports a critical role of the immune system in AMD^3,4^. Recruitment of microglial cells, the immunocompetent cells of the central nervous system (CNS), has been associated with the progression and severity of AMD^5,6^. Indeed, microglia reactivity is involved in the recruitment and activation of complement system^7,8^ and inflammasome pathways^9^, which may contribute to photoreceptor degeneration^10^.

The complement system and inflammasome pathways, key innate immune defenses against inflammation, orchestrate critical responses in the CNS. Several studies reported the involvement of the complement cascades in both acute and chronic disease conditions^11,12^. Genetic variations of several complement-related genes are associated with AMD pathogenesis^13–16^. Similarly, elevated levels of complement factors have been detected in the plasma and aqueous humor of AMD patients^17,18^. Moreover, complement proteins are present in drusen. Notably, A2E, a RPE lipofuscin and drusen component, triggers microglia reactivity and complement activation by increasing complement factor B (CFB) and decreasing complement factor H (CFH) expression, leading to photoreceptor degeneration^8^.

Inflammasome is a large multiprotein complex that takes part in the innate immune response, promoting interleukin 1β (IL-1β) and interleukin 18 (IL-18) maturation, through caspase-1-mediated cell death, known as pyroptosis^19^. AMD-associated inflammasome activation has been widely described. There is evidence showing the ability of drusen to induce the activation of inflammasome, namely NLRP3 inflammasome, in retinal mononuclear cells^20^. Inflammasome has been detected in the RPE, in both dry and wet AMD, and might contribute to RPE degeneration^21,22^. Interestingly, factors released from reactive microglia can also induce the activation of NLRP3 inflammasome in ARPE-19 cells^9^.

Adenosine is a crucial neuromodulator in the CNS, and blockade of adenosine A_2A_ receptor (A_2A_R) affords robust neuroprotection against different neurodegenerative conditions^23^. We have recently reported that A_2A_R blockade prevents retinal microglia reactivity and the associated neuroinflammatory response, conferring protection to retinal ganglion cells, in experimental models of glaucoma^24–26^. Still, the impact of A_2A_R modulation on the complement system and its potential beneficial effects on AMD remain to be elucidated.

Hence, the main aim of this work was to evaluate the outcome of A_2A_R blockade in the microglial complement system. Moreover, we evaluated the potential beneficial effects of blocking A_2A_R in human microglial cells on the inflammatory response of ARPE-19 cells, inflammasome activation and photoreceptor loss.

## Results

### Inflammatory stimuli increase the release of adenosine and the density of A2AR in human microglial cells

It is well known that A_2A_R is up-regulated under noxious conditions^24,25,27^. Therefore, we firstly investigated whether the incubation of human microglial cells with Zymosan and phorbol myristate acetate (PMA), two potent pro-inflammatory stimuli^28^, would be able to increase the levels of extracellular adenosine, which then could boost A_2A_R activation, and affect A_2A_R expression.

**Figure 1:**
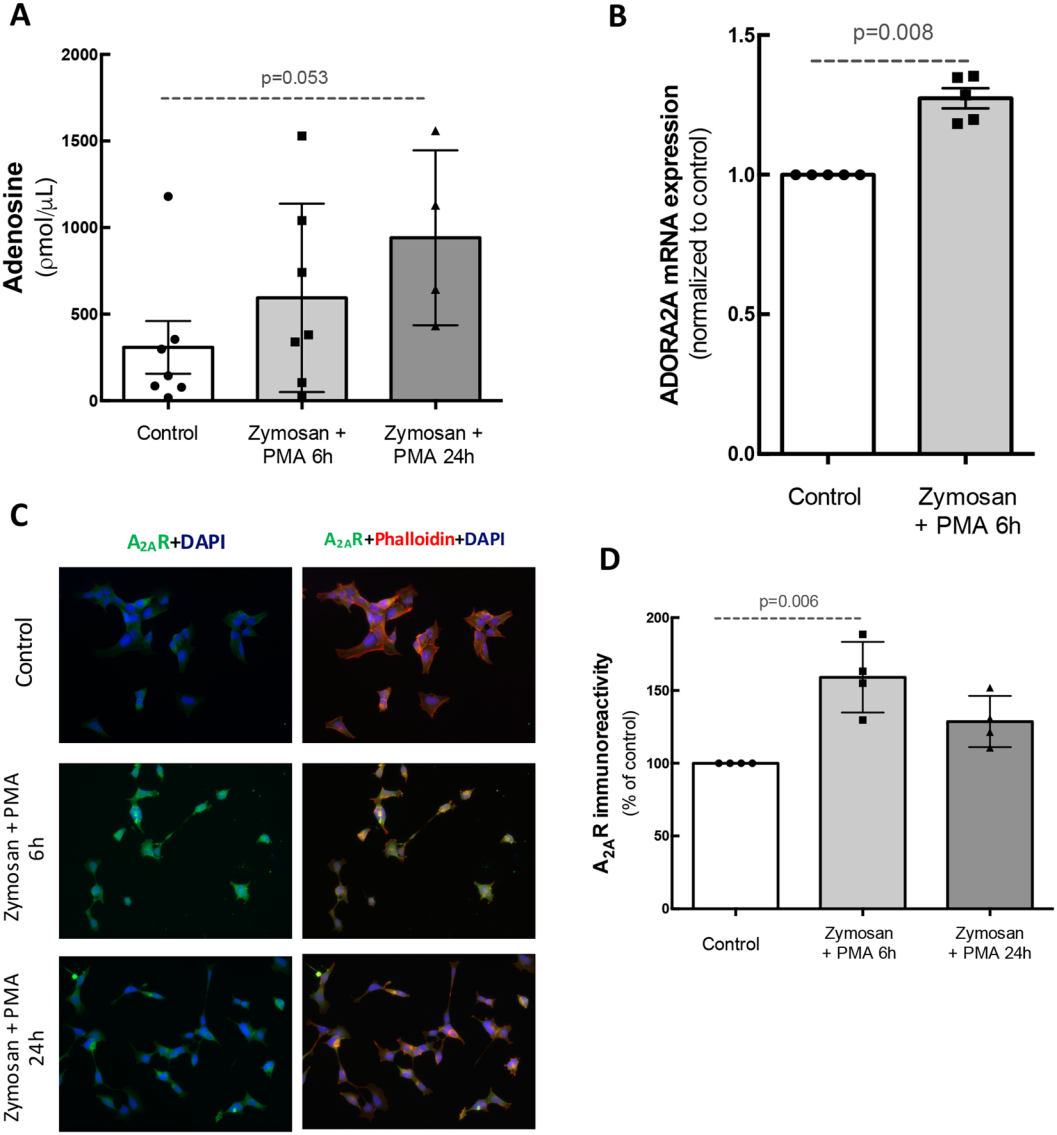
Inflammatory stimulus increases the release of adenosine and the expression and density of A_2A_R in human microglia. Human immortalized microglial cells were challenged with Zymosan + PMA for 6 h or 24 h. **(A**) Adenosine concentration in culture supernatants. Data are expressed as percentage of control and represent the mean ± SEM of 3–7 independent experiments (presented with the scatterplot with the individual data points). **(B)** The mRNA levels of A_2A_R were assessed by qPCR relatively to *hprt* (housekeeping gene). Results are normalized to control from 3–7 independent experiments. Mann-Whitney test. **(C)** Microglial cells (red; phalloidin) were immunostained for A_2A_R (green). Nuclei were stained with DAPI (bue). Images are representative of 4–7 independent experiments. **(D)** Densitometric analysis of A_2A_R immunoreactivity. Data represent the mean ± SEM, with the scatterplot with the individual data points. Results are expressed as percentage of control and were obtained from 4–7 independent experiments. Kruskall-Wallis test, followed by Dunn’s multiple comparison test.

In control conditions, the concentration of extracellular adenosine was 308 ± 152 pmol/μL (n = 7). The incubation with Zymosan + PMA for 6 h or 24 h increased the concentration of adenosine to 593.7 ± 205.4 or 941.1 ± 205 ρmol/μL, respectively (n = 4–7; Fig. 1A). Also, A_2A_R mRNA expression was significantly up-regulated (1.3-fold increase, n = 5) after 6 h incubation with Zymosan + PMA when compared with the control condition (Fig. 1B). Moreover, the incubation of human microglia with Zymosan + PMA for 6 h or 24 h increased the immunoreactivity of A_2A_R (159 ± 12.1 and 128.6 ± 8.7% of control, respectively; n = 4; Fig. 1C and D). These results demonstrate that pro-inflammatory conditions can trigger an increase in the release of adenosine from human microglia that may then signal through the upregulated A_2A_R.

### Treatment with an A_2A_R antagonist reduces the inflammatory response in human microglia

Considering that A_2A_R expression and density increased in human microglia exposed to Zymosan + PMA and that a selective antagonist of A_2A_R prevented the neuroinflammatory response^24^, we next investigated whether A_2A_R blockade could influence human microglia reactivity.

**Figure 2:**
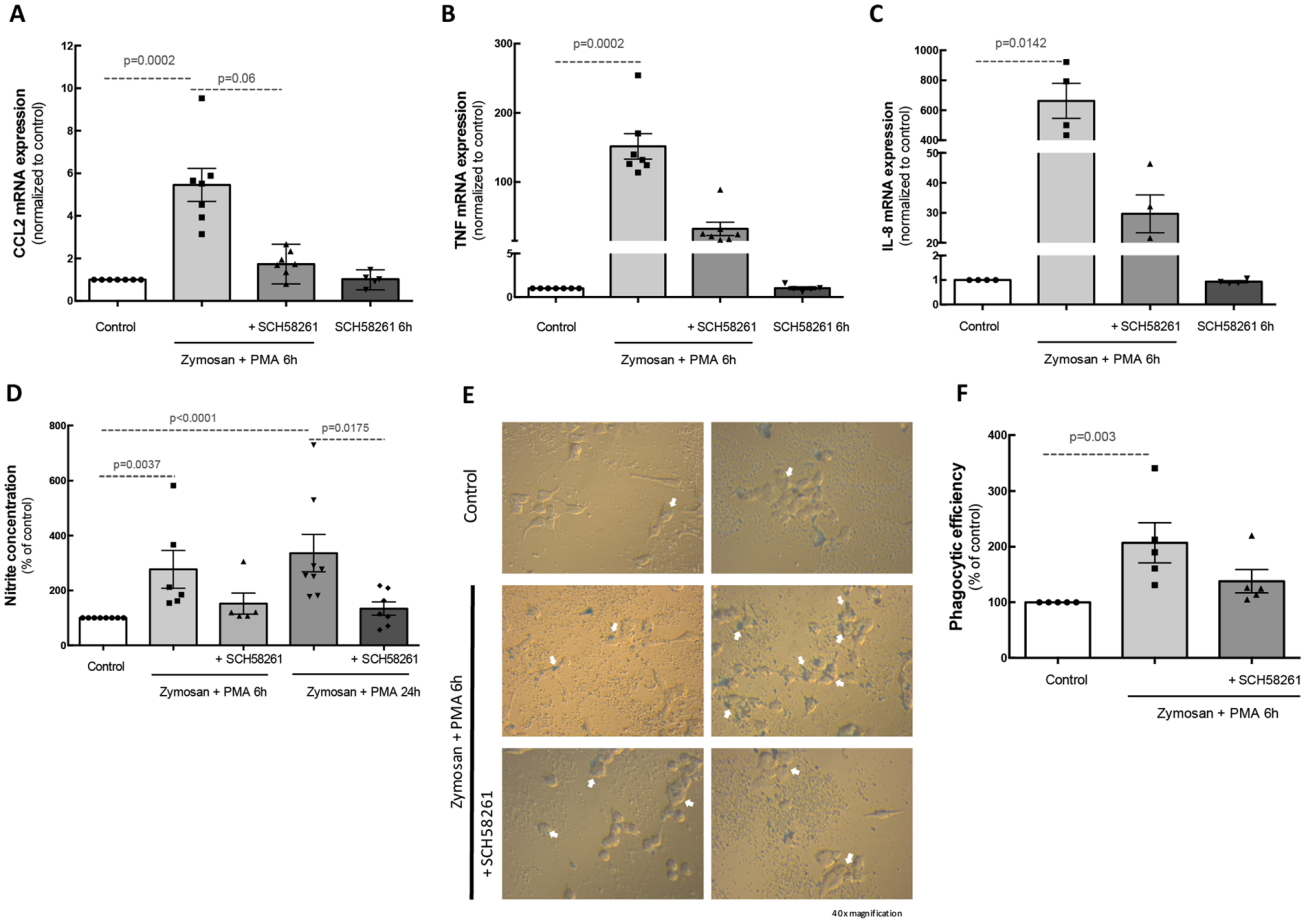
A_2A_R blockade reduces the inflammatory response in human microglia. Human microglial cells were challenged with Zymosan + PMA in the absence or presence of 50 nM SCH58261. The mRNA levels of CCL2 **(A)**, TNF **(B)** and IL-8 **(C)** were assessed by qPCR. Results are normalized to control from 5–7 independent experiments. **(D)** The release of NO was assessed by the Griess reaction assay in culture supernatants. Results are expressed in percentage of control and presented as the mean ± SEM of 4–7 independent experiments (the individual data points for each condition are also presented). **(E)** Human microglial cells were incubated with Zymosan + PMA for 2 h in the absence or presence of SCH58261, before adding polystyrene beads for 6 h (white arrows). Images were acquired and the phagocytic efficiency was determined **(F)**. Results are expressed as percentage of control and presented as the mean ± SEM of 5 independent experiments (the individual data points for each condition are also presented). Kruskall-Wallis test, followed by Dunn’s multiple comparison test.

The incubation of human microglia with Zymosan + PMA for 6 h up-regulated the mRNA expression of the inflammatory markers CCL2, TNF and IL-8 (n = 7). Pre-treatment with 50 nM SCH58261, the A_2A_R antagonist, reduced the stimulatory effect of Zymosan + PMA (n = 7; Fig. 2A–C). SCH58261 by itself did not alter the expression of these pro-inflammatory markers.

The incubation of microglia with Zymosan + PMA for 6 h or 24 h also increased the release of NO by human microglia, as determined by the nitrite concentration in culture supernatants (223.9 ± 37.1 and 298.3 ± 63.3% of control, respectively, n = 6–8). The blockade of A_2A_R prevented the increase in NO induced by Zymosan + PMA (118.7 ± 12.3 and 116.8 ± 20.8% of control for 6 h and 24 h, respectively) (Fig. 2D).

Since reactive microglia are characterized by the ability to phagocytose foreign material, we determined the phagocytic efficiency of human microglia following exposure to Zymosan + PMA, in the absence or presence of the A_2A_R antagonist. The incubation of human microglia with Zymosan + PMA significantly increased the number of phagocytosed polystryrene microparticles (206.9 ± 36% of control, n = 5). This effect was partially reduced by the pre-treatment with SCH58261 (138 ± 20.6% of control, n = 5). Taken together, these results indicate that A_2A_R blockade prevents the shift of human microglia into a reactive phenotype.

### Blockade of A_2A_R prevents the upregulation of complement components in human microglia induced by Zymosan + PMA

Complement system modulation is an emerging therapeutic strategy for AMD^29^. Although A_2A_R blockade controls microglia reactivity^23^, the direct effects of A_2A_R blockade on the complement system of microglia are unknown. Hence, we addressed this question in human immortalized microglial cells to evaluate whether the A_2A_R antagonist could modulate the expression of complement factor in microglial cells.

**Figure 3:**
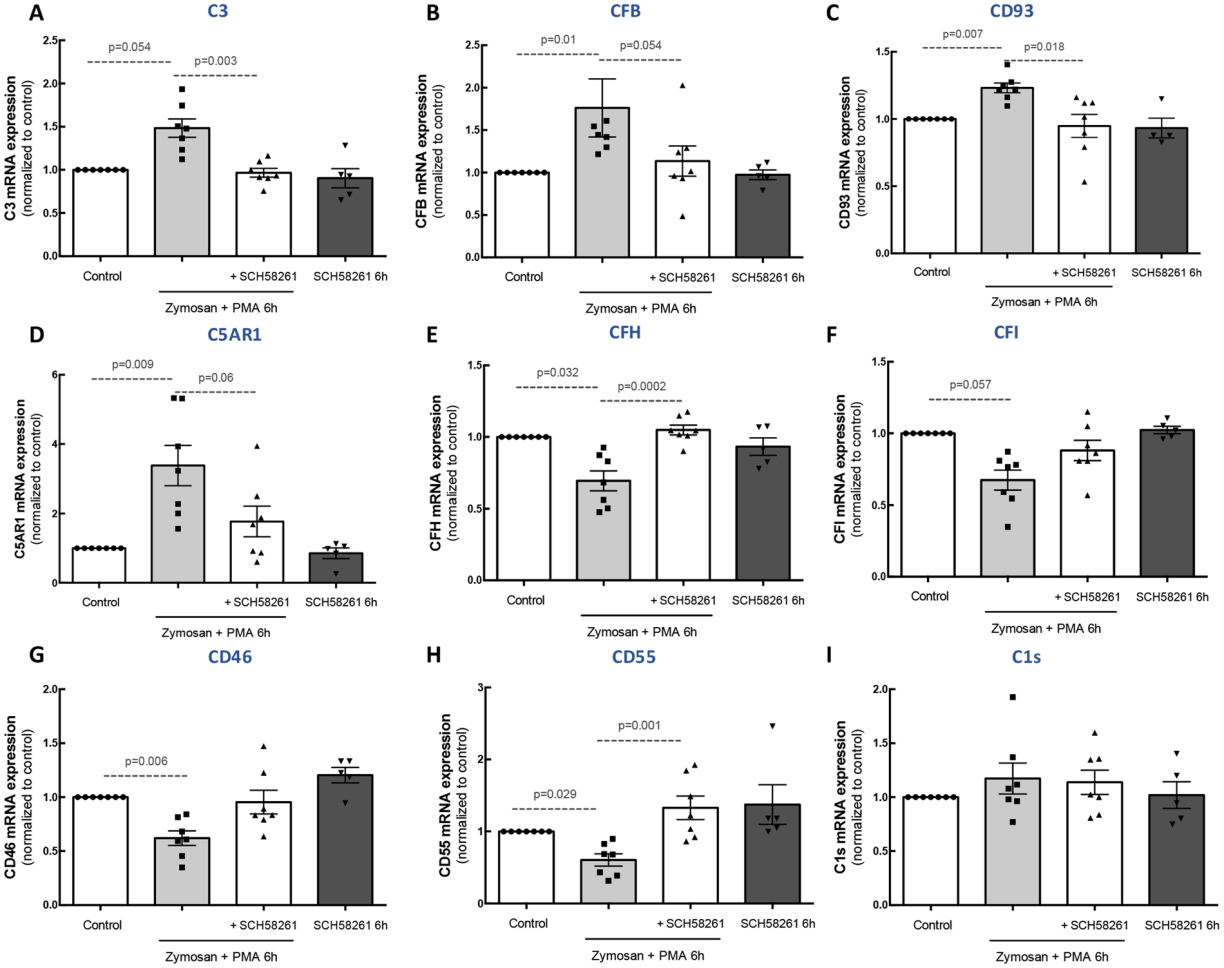
Blockade of A_2A_R prevents the alterations in complement cascade components in reactive human microglial cells. Human microglial cells were challenged with Zymosan + PMA in the presence or absence of 50 nM SCH58261. The expression levels of complement cascade components C3 **(A)**, CFB **(B)**, CFH **(C)**, CFI **(D)**, CD93 **(E)**, C1s **(F)**, C5AR1 **(G)**, CD46 **(H)** and CD55 **(I)** were assessed by qPCR. Results are normalized to control and presented as the mean ± SEM of 5–7 independent experiments (the individual data points for each condition are also presented). Kruskall-Wallis test, followed by Dunn’s multiple comparison test.

Incubation of human microglia with Zymosan + PMA for 6 h increased the expression of complement components associated with the activation of the cascade: complement C3 (Fig. 3A), CFB (Fig. 3B), CD91 (also known as C1qr; Fig. 3C), and complement C5a receptor 1 (C5AR1) (Fig. 3D). When the cells were pre-treated with the A_2A_R selective antagonist, the upregulation of these components was prevented. Concerning the inhibitory complement components, Zymosan + PMA decreased CFH (Fig. 3E), CFI (Fig. 3F), CD46 (also known as MCP; Fig. 3G), and CD55 (also known as DAF; Fig. 3H), and these inhibitory effects were absent in the presence of the A_2A_R antagonist. No alterations were observed in the mRNA levels of C1s (Fig. 3I). Treatment of human microglia with SCH58261 did not change the expression of complement components.

### A_2A_R blockade prevents microglia reactivity induced by exposure to ARPE-19 debris

Drusen components can induce microglia reactivity and complement activation^8^. Therefore, we evaluated whether A_2A_R blockade could prevent the reactivity of human microglia induced by RPE debris, which were used to mimic drusen components.

We first evaluated the ability of different concentrations and times of exposure of ARPE-19 debris to induce an inflammatory response in microglial cells (Sup. Figure 1). All challenges with ARPE-19 cell debris induced up-regulation of CCL2 and IL-8 mRNA expression, but not TNF. We choose 5 μg/μL ARPE-19 cell debris since it was the shorter time point and lower concentration.

**Figure 4:**
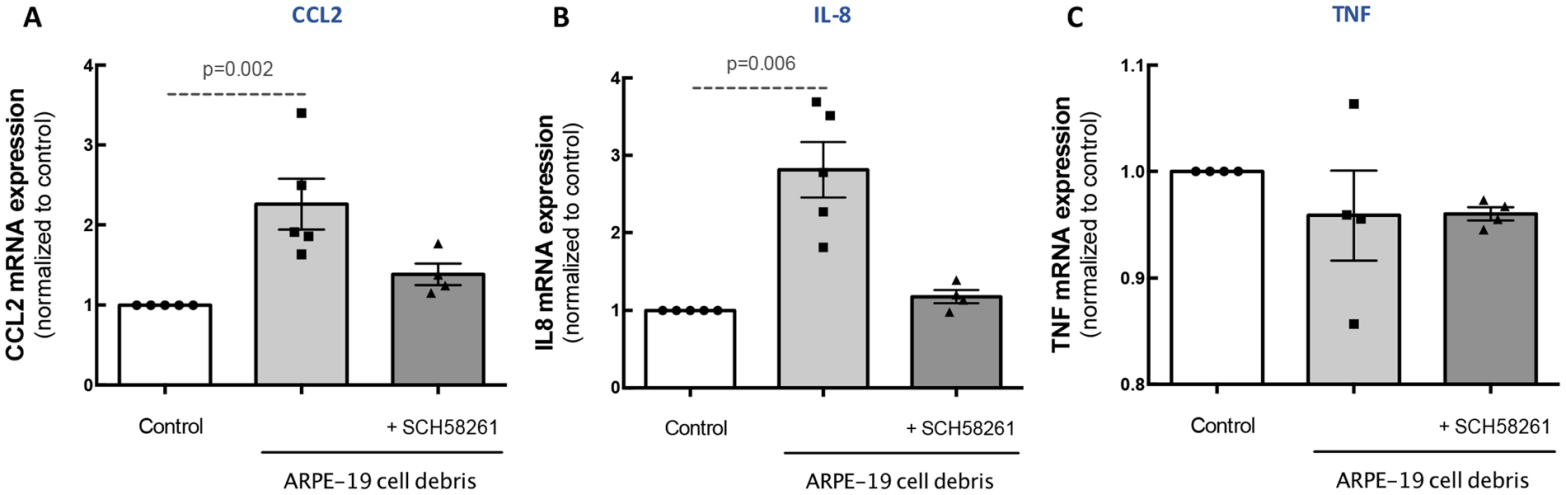
A_2A_R blockade prevents ARPE-19 debris-induced microglia inflammatory response. Human microglia were exposed to 5 µg ARPE-19 apoptotic cell debris for 6 h in the absence or presence of the A_2A_R antagonist. The mRNA expression levels of CCL2 **(A)**, TNF **(B)** and IL-8 **(C)** were assessed by qPCR. Results are normalized to control and presented as the mean ± SEM of 5 independent experiments (the individual data points for each condition are also presented). Kruskall-Wallis test, followed by Dunn’s multiple comparison test.

Then, human microglial cells were challenged with RPE debris (5 μg/μL of ARPE-19 cell debris for 6 h) 45 minutes after incubation with A_2A_R antagonist. The A_2A_R antagonist partially prevented the increase in the mRNA expression of CCL2 (Fig. 4A) and IL-8 (Fig. 4B). The mRNA expression levels of TNF were not altered by ARPE-19 cell debris in the absence or presence of SCH58261 (Fig. 4C).

### A_2A_R blockade prevents changes in complement components in microglia induced by exposure to ARPE-19 debris

**Figure 5:**
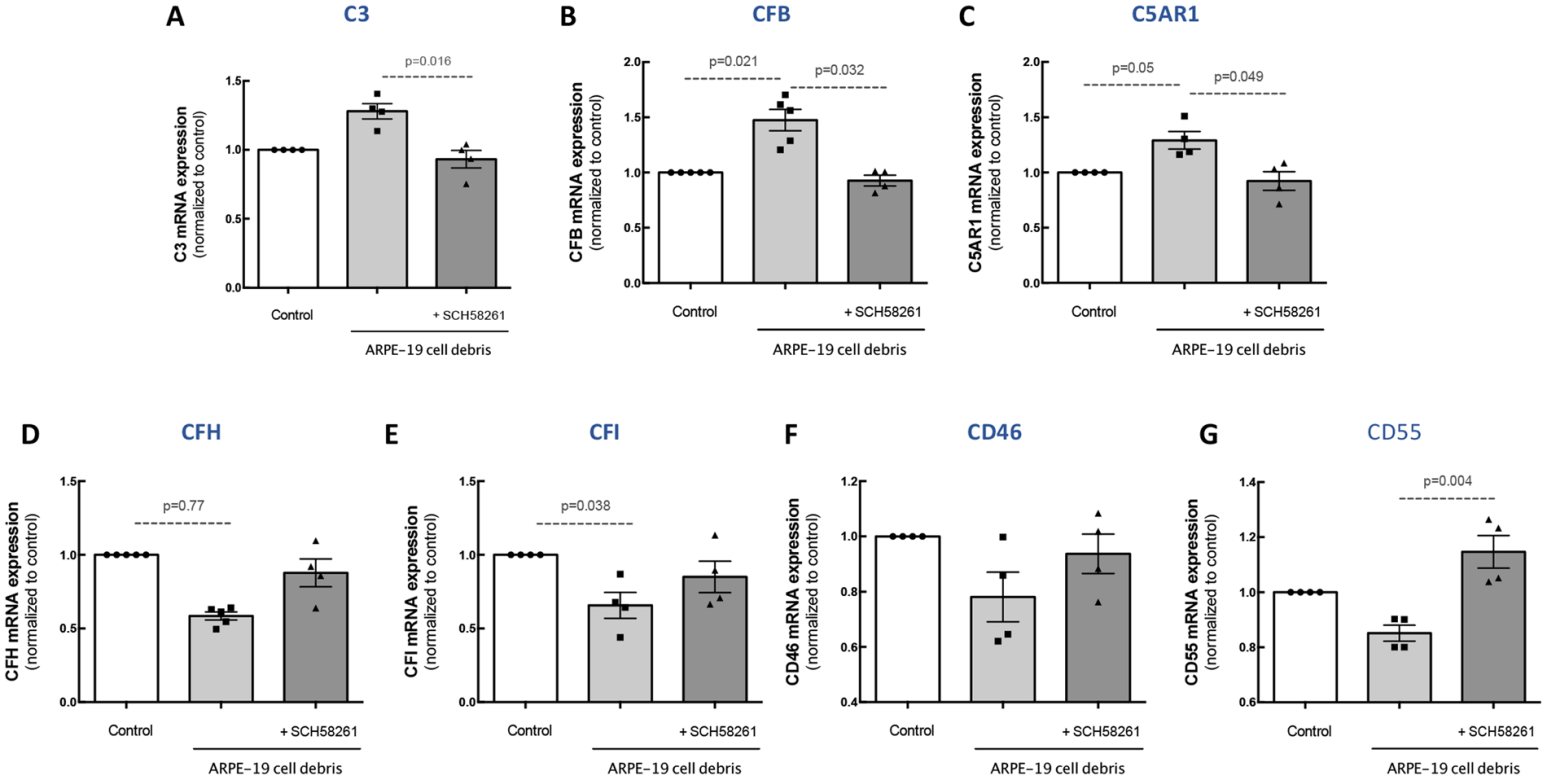
A_2A_R blockade prevents microglia complement cascade alterations induced by ARPE-19 debris. Human microglia were exposed to 5 µg/mL ARPE-19 apoptotic cell debris for 6 h in the presence or absence of A_2A_R antagonist. The mRNA expression levels of complement cascade components C3 **(A)**, CFB **(B)**, CFH **(C)**, CFI **(D)**, C5AR1 **(E)**, CD46 **(F)** and CD55 **(G)** were assessed by qPCR. Results are normalized to control and presented as the mean ± SEM of 5 independent experiments (the individual data points for each condition are also presented). Kruskall-Wallis test, followed by Dunn’s multiple comparison test.

We then evaluated whether the ARPE-19 cell debris were able to induce alterations in the expression of complement components of human microglia. When human microglia were incubated for 6 h with ARPE-19 cell debris the mRNA expression of C3 (Fig. 5A; n = 5), CFB (Fig. 5B; n = 5) and C5AR1 (Fig. 5C; n = 5) increased and this effect was prevented by the blockade of A_2A_R. The transcript levels of the inhibitory components CFH (Fig. 5D), CFI (Fig. 5E), CD46 (Fig. 5F) and CD55 (Fig. 5G) in human microglia presented a tendency to decrease upon exposure to ARPE-19 cell debris (n = 5). Pre-treatment with SCH58261 partially prevented these alterations (n = 5).

### The blockade of A_2A_R in microglia prevents an inflammatory response and complement activation in ARPE-19 cells induced by exposure to microglia conditioned medium

We have previously reported that the exposure of ARPE-19 cells to conditioned medium from reactive microglia increases the expression of pro-inflammatory markers^9^. In addition, we demonstrated that the blockade of A_2A_R prevents retinal inflammation^24,25^. Therefore, we hypothesized that if A_2A_R blockade halts microglia reactivity, this would be sufficient to limit RPE responses to microglia-conditioned medium (MCM).

**Figure 6:**
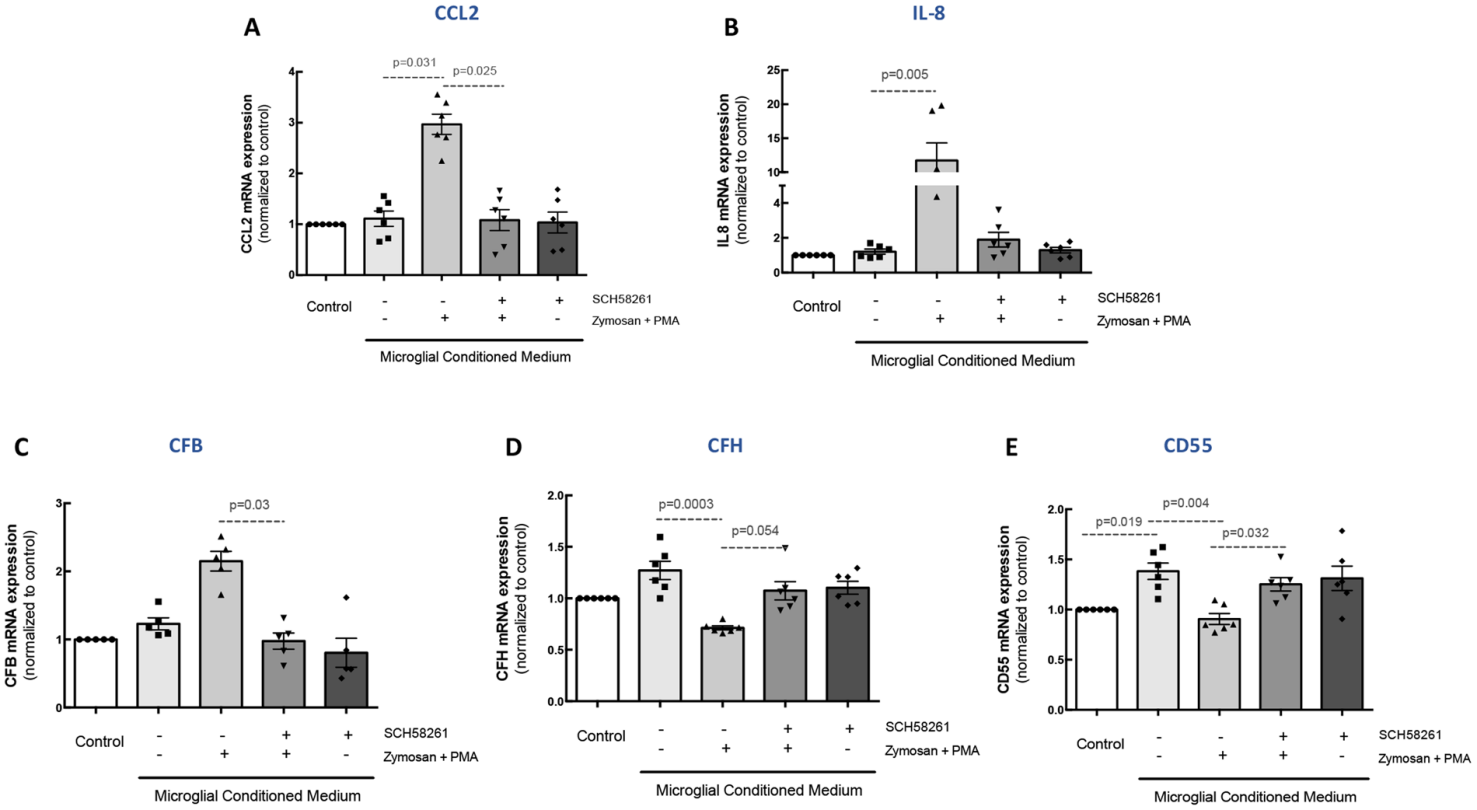
Blockade of A_2A_R in microglia prevents the inflammatory response and complement cascade alterations in ARPE-19 cells. Microglia were pretreated with 50 nM SCH58261 and then challenged with Zymosan + PMA for 6 h. MCM was added to ARPE-19 cells for 48 h. The mRNA expression of CCL2 **(A)** IL-8 **(B)**, CFB **(C)**, CFH **(D)**, and CD55 **(E)** was assessed by qPCR. Results are normalized to control and presented as the mean ± SEM of 6 independent experiments (the individual data points for each condition are also presented). Kruskall-Wallis test, followed by Dunn’s multiple comparison test.

The mRNA expression of CCL2 and IL-8 (Fig. 6A and B) was up-regulated in ARPE-19 cells (2.98- and 11.7-fold increase comparing with the control MCM; n = 6) after exposure to MCM from Zymosan + PMA-treated cells. The incubation of ARPE-19 cells with cell culture supernatants from microglia pre-treated with A_2A_R antagonist before Zymosan + PMA exposure did not change the expression of CCL2 and IL-8, compared with the control.

The exposure of ARPE-19 cells to MCM from cells exposed to Zymosan + PMA increased the mRNA expression of CFB (2.15-fold increase relative to control MCM; n = 6; Fig. 6C) and downregulated CFH and CD55 mRNA expression (0.71- and 0.9-fold change, respectively; Fig. 6D and E), suggesting the activation of the complement cascade in ARPE-19 cells. The A_2A_R blockade in microglial cells prevented the alterations in CFB, CFH, and CD55 detected in RPE cells. Also, MCM obtained from microglial cells exposed to SCH58261 did not alter the expression of these markers. In order to exclude a potential carryover effect, ARPE-19 cells were directly incubated with Zymosan + PMA, but no changes in the mRNA expression of these markers were detected (Supplementary Fig. 2). Importantly, ARPE-19 cells are not immunoreactive for A_2A_R (Supplementary Fig. 3) and do not express A_2A_R mRNA (data not shown).

### A_2A_R blockade in microglia prevents inflammasome activation in RPE cells

We have recently described a role for reactive microglia in the activation of RPE inflammasome^9^. Therefore, we investigated whether blocking A_2A_R in microglia could prevent the activation of inflammasome in ARPE-19 cells triggered by exposure to MCM collected from microglia cultures exposed to pro-inflammatory conditions.

**Figure 7:**
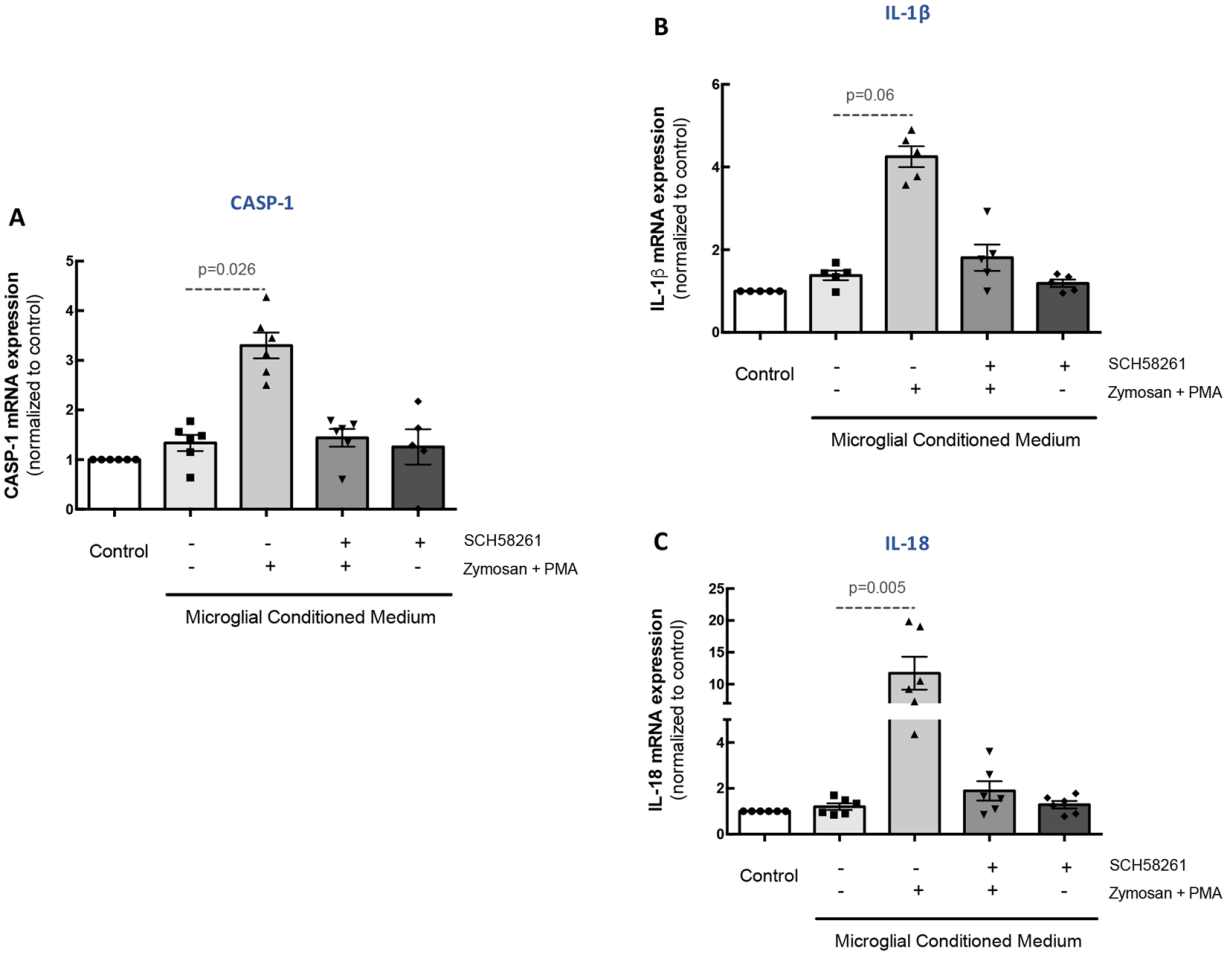
A_2A_R blockade in reactive human microglia prevents inflammasome activation in ARPE-19 cells. Microglia were pretreated with 50 nM SCH58261 and then challenged with Zymosan + PMA for 6 h. MCM was added to ARPE-19 cells for 48 h. The mRNA expression of caspase-1 **(A)** IL-1β **(B)**, and IL-18 **(C)** was assessed by qPCR. Results are normalized to control and presented as the mean ± SEM of 6 independent experiments (the individual data points for each condition are also presented). Kruskall-Wallis test, followed by Dunn’s multiple comparison test.

When ARPE-19 cells were exposed to MCM from non-stimulated microglial cell cultures, no significant alterations were detected in the expression of several components (caspase-1, IL-1β and IL-18) of the inflammasome pathway components of ARPE-19 cells. However, in ARPE-19 cells exposed to MCM from microglial cell cultures exposed to Zymosan + PMA there was an up-regulation of the mRNA expression levels of caspase 1 (CASP1; Fig. 7A), IL-1β (Fig. 7B) and IL-18 (Fig. 7C). Importantly, pre-treatment of human microglia with the A_2A_R antagonist prevented this effect (Fig. 7). MCM obtained from microglial cells exposed to SCH58261 did not alter the expression of these markers.

### Blockade of A_2A_R increases microglia clearance of photoreceptor apoptotic debris

**Figure 8:**
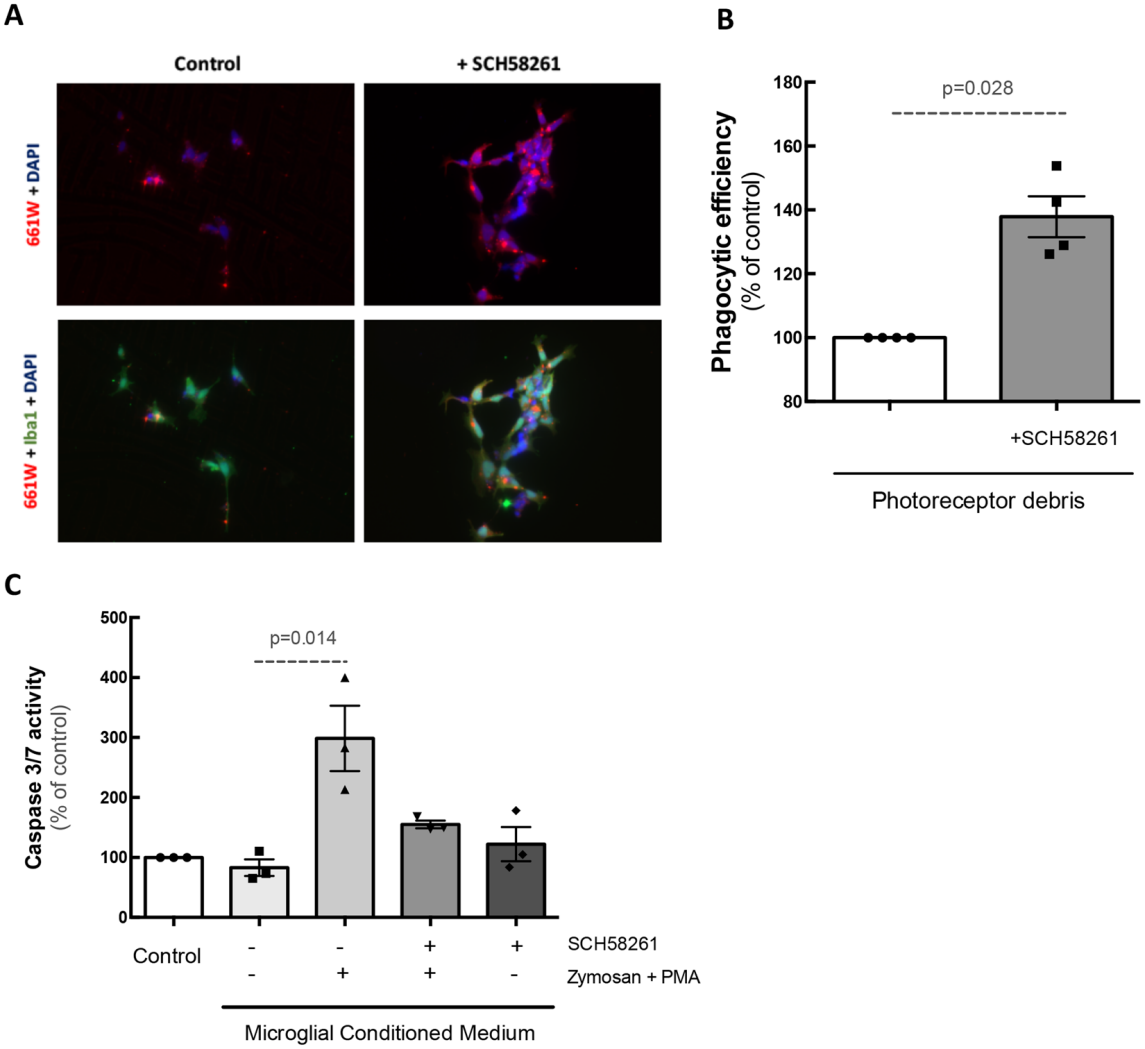
Blockade of A_2A_R increases the clearance of apoptotic photoreceptors debris by human microglial cells and prevents microglia-mediated increase of photoreceptor apoptosis. Human microglial cells (green) were exposed to CellTracker CM-DiI-labelled photoreceptors (red) in the absence or presence of SCH58261 for 6 h. Nuclei were counterstained with DAPI (blue). Representative images are depicted in (**A**) and the phagocytic efficiency was determined (**B**). Results are expressed as percentage of control and presented as the mean ± SEM of 4 independent experiments (the individual data points for each condition are also presented). (**C**) Photoreceptors were exposed to microglia conditioned medium for 48 h and the activity of caspase 3/7 was determined. Results are expressed as percentage of control and presented as the mean ± SEM of 3 independent experiments (the individual data points for each condition are also presented). Kruskall-Wallis test, followed by Dunn’s multiple comparison test.

An effective phagocytic clearance of photoreceptor apoptotic debris by microglia is fundamental for the maintenance of retinal integrity^30,31^. Hence, we next investigated the impact of A_2A_R blockade on the clearance of photoreceptor debris by microglia. For this purpose, control or Zymosan + PMA-treated human microglia, in the absence or presence of SCH58261, were exposed to the 661 W photoreceptor cell line apoptotic debris and the phagocytic efficiency was determined (Fig. 8A). Blockade of A_2A_R significantly increased the phagocytic efficiency of microglia (137.8 ± 6.1% of non-treated, n = 4; Fig. 8B), suggesting an increase in the clearance of apoptotic material from photoreceptors.

### Control of microglia reactivity by the A_2A_R antagonist prevents 661 W photoreceptor cell death

In order to further explore the influence of blocking A_2A_R on microglial-induced neurotoxicity, 661 W photoreceptor-like cells were incubated for 48 h with untreated or Zymosan + PMA-treated MCM, in the absence or presence of SCH58261. Thereafter, the activity of caspases 3/7 was determined to estimate the apoptotic activity in 661 W cells (Fig. 8C). 661 W photoreceptors cultured with MCM from reactive cells showed increased caspase 3/7 activity (289.5 ± 54% of the control, n = 3). The activity of caspase 3/7 was significantly reduced when microglia were pre-treated with SCH58261 (115.6 ± 6% of control; n = 3), suggesting that the control of microglia reactivity due to A_2A_R blockade protects against the neurotoxic effects of microglia.

## Discussion

**Figure 9:**
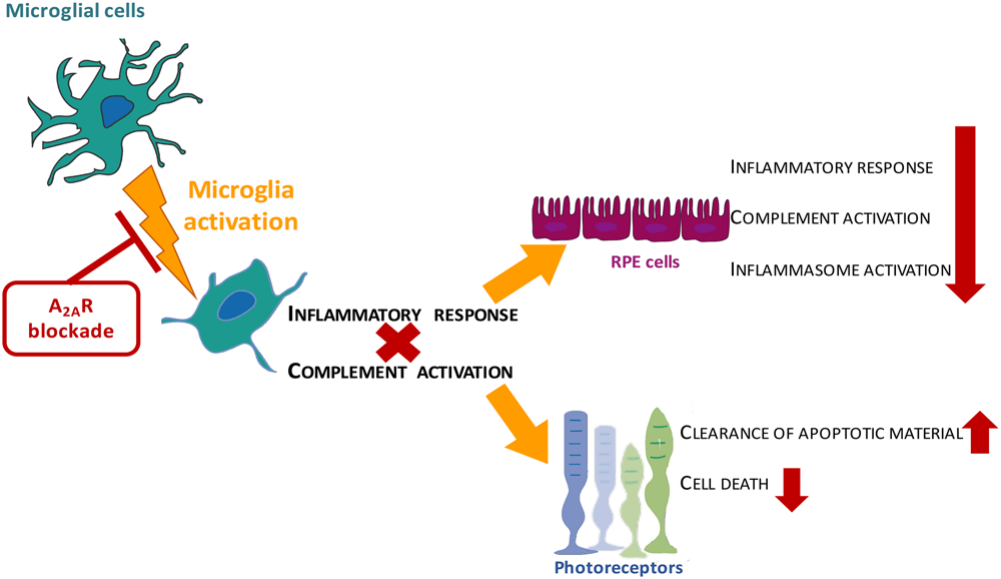
The blockade of A_2A_R in microglial cells prevent the loss of photoreceptors and reduced RPE dysfunction. Changes in homeostasis, trigger microglia reactivity that up-regulate inflammatory mediators and activate the complement cascade. This induce an inflammatory response is accompanied by complement and inflammasome activation on RPE cells and increased photoreceptor cell death. When microglial cells are treated with a selective antagonist of A_2A_R, the microglial immune response is reduced (effects indicated with red symbols). Importantly, the blockade of A_2A_R in microglia reduces the inflammatory response in RPE cells, as well as the activation of the complement cascade and inflammasome. Likewise, blocking A_2A_R in microglia reduces photoreceptor cell death elicited by exposure to the supernatants of reactive microglia and increases the clearance of photoreceptor apoptotic material.

In the present work, using *in vitro* models, we show for the first time that the blockade of A_2A_R in microglia impacts the expression of the complement system components in microglial cells. Importantly, the blockade of A_2A_R in microglia also reduces the inflammatory response in RPE cells, as well as the activation of the complement cascade and inflammasome. Likewise, blocking A_2A_R in microglia reduces photoreceptor cell death elicited by exposure to the supernatants of reactive microglia and increases the clearance of photoreceptor apoptotic material (Fig. 9).

Increased expression of A_2A_R, as occurs in retinal microglia^24^, has been associated with several CNS noxious conditions^23,32^. It has been shown that activation of A_2A_R promotes microglia-mediated inflammatory responses, by potentiating NO release by activated microglia^33^. Here, by exposing human microglial cells to Zymosan and PMA, known to induce microglia activation by mimicking pathogenic infection^28,34^, we report an increase in the concentration of extracellular adenosine as well as in the expression of A_2A_R in these cells, suggesting that A_2A_R signaling might be boosted in microglia thus promoting inflammation.

Parallel to what we have previously described for rat microglia^24^, blockade of A_2A_R in human microglial cells prevents the reactive microglia phenotype, as indicated by the decrease in the expression of inflammatory mediators (CCL2, TNF and IL-8) and the reduced release of NO and phagocytic efficiency. Importantly, the exposure of human microglia to the apoptotic human ARPE-19 cell debris that resemble drusen material^35^ also up-regulated the expression of inflammatory mediators (CCL2 and IL-8), which was also prevented by the blockade of A_2A_R. Interestingly TNF expression was increased in microglia incubated with Zymosan + PMA but not with ARPE-19 debris, which suggests that different signaling pathways are elicited by the different noxious conditions. Several signaling pathways have been demonstrated to regulate TNF expression, such as nuclear factor kappa B (NF-κB), as well as members of the mitogen activated protein kinase family, including ERK1/2 (extracellular receptor activated kinases 1/2), p38 and c-jun N-terminal kinase (JNK)^36^. Future studies can focus on the signaling pathways activated in each condition.

Exposure of human microglia to an inflammatory stimulus or to ARPE-19 cell debris also induced the activation of the complement cascade by up-regulating the expression of C3, CFB, C1q and C5AR1 and down-regulating CFH, CFI, CD46 and CD93. Previous studies reported that the up-regulation of C3 in microglia is associated with the pathogenesis of age-related retinal diseases^7,37,38^. Furthermore, when exposed to A2E, a drusen component, microglial cells up-regulate the expression of CFB and down-regulate the levels of CFH, a process that has been associated with photoreceptor loss^8^. Likewise, increased plasma levels and aqueous humor concentrations of complement components in AMD patients have been associated with low responsiveness to current therapies^17,18^. Therefore, supplemental therapies targeting complement system activation have been suggested as novel strategies for AMD management^17,29,39^. The selective antagonist of A_2A_R was able to prevent the alterations occurring in the complement system components in reactive human microglia, suggesting that A_2A_R not only modulates microglia pro-inflammatory responses, but can also modulate the activation of complement cascade.

Several studies have suggested a crosstalk between signals arising from reactive microglial cells and RPE that regulate the expression and secretion of pro-inflammatory, chemotactic, and pro-angiogenic molecules^9,40,41^. This crosstalk may be also associated with drusen formation as a product of local inflammatory responses^42^. Here we show that reactive microglial cells may also trigger the activation of the complement system in ARPE-19 cells, further corroborating the role of microglia on RPE dysfunction. Again, the blockade of A_2A_R in microglia had the ability to prevent the activation of the complement system in RPE, suggesting that the control of microglia reactivity is sufficient to prevent microglia-induced effects on RPE.

NLPR3 inflammasome activation in the RPE has been described in AMD models^21,43,44^ and has been proposed to be a causal factor for RPE dysfunction and degeneration^45^. In accordance with our recent report^9^, we detected increased expression of caspase-1, IL-1β and IL-8 in ARPE-19 cells, supporting the idea that reactive microglial cells release factors that induce the activation of NLRP3 inflammasome in ARPE-19 cells. Remarkably, when microglial cells were treated with SCH58261, the activation of inflammasome in ARPE-19 was abolished, suggesting that the blockade of A_2A_R in microglia modulates inflammasome activation in RPE. In macrophages, adenosine modulates the inflammasome activation through A_2A_R^46^, which is in accordance with our data, where we found that blocking this receptor prevents the up-regulation of NLRP3 and IL-18 in reactive microglial cells (Supplementary Figure 4). However, there were no previous reports about the modulation of RPE inflammasome by A_2A_R. Also, we have previously described that reactive microglia-released material might induce activation of autophagy in RPE^9^. Although we have not evaluated the role of microglial A_2A_R blockade on the ARPE-19 autophagic pathways, one can speculate that autophagy might also be impacted, since autophagy regulates NLRP3 inflammasome activation^47,48^. Indeed, future studies should focus on understanding a possible role of A_2A_R modulation in autophagy, which has been shown to have a fundamental role on the onset of AMD, as it modulates RPE inflammasome activation^49–51^ and RPE cell death^52,53^.

Photoreceptor cell loss by apoptosis is a major feature of AMD^54^, and an effective microglia-mediated clearance of the apoptotic cells is considered to be associated with an anti-inflammatory phenotype^55^. Indeed, it has been shown that photoreceptor proteins are able to induce microglia phagocytic activity and promote the inflammatory response^56^. Notably, the selective A_2A_R antagonist was able to increase the phagocytosis of photoreceptor debris by microglia, suggesting that blocking A_2A_R appears to have additional beneficial effects, such as increased clearance of the apoptotic material. Also, a more efficient clearance of the apoptotic material might contribute to a reduction of the inflammatory response triggered by the exposure to photoreceptor proteins. In this work we demonstrated that the A_2A_R antagonist reduces the phagocytosis of polystyrene beads by microglia, while it increased the phagocytosis of apoptotic cells. These apparent contradictory results operated by A_2A_R may imply molecular signals that induce distinct responses in microglia: it promotes the clearance of dead cells probably to halt the inflammatory response but when microglia face inert material, such as polystyrene beads, microglia phagocytose less when treated with SCH58261.

In fact, the crosstalk between microglial cells and photoreceptors is indubitable, since factors present in the culture medium of reactive microglial cells are able to induce photoreceptor cell apoptosis^57–59^. Importantly, blockade of microglial A_2A_R is also able to reduce this outcome, suggesting that modulation of microglial cells reactivity by A_2A_R blockade can afford protective effects against photoreceptor cell death.

The role of inflammation and particularly the role of retinal microglia reactivity in the pathophysiology of AMD is unquestionable. Moreover, microglia-RPE crosstalk have long been associated with AMD pathogenesis, being speculated that this crosstalk can act as a positive feedback perpetuating the inflammatory response and sub-retinal microglia accumulation, fostering the proper mechanisms that promote AMD-related retinal dysfunction^40^. Complement proteins^8,37^, IL-1β^60^ and NF-κB^10,57^ have been already proposed as microglial modulatory factors that can in turn modulate the RPE inflammatory response being involved in photoreceptor loss. Still, novel therapeutic strategies aiming diminishing the effects of these mechanisms are required to reduce immune response in the context of AMD. Noteworthy, the control of the complement system has been identified as a possible therapeutic strategy for managing AMD^29^, but some concerns might be raised relatively to its efficacy. Recently, ROCHE announced that the clinical trial (phase IIB) for Lampalizumab (NCT01602120), an antibody-binding fragment of a humanized monoclonal antibody that binds to complement factor D, which has been implicated in the pathogenesis of geographic atrophy^61^, did not reduced the geographic atrophy lesion area at 1 year of treatment^62^.

In this study, the different cell lines allowed us to dissect the role of A_2A_R blockade in microglial cells, the immune cells of the CNS, without the contribution of infiltrating immune cells, and the possible crosstalk between microglia, RPE cells and photoreceptors. Although it is important to refer that despite having some advantages over primary cells, cell lines have some limitations because they do not always accurately replicate the features of native cells in the tissue. Indeed, ARPE-19 cell line is one of the most commonly used cell lines for the study of RPE. However, these cells exhibit particularities different from RPE cells in their native environment, such as reduced transepithelial resistance, reduced levels of secreted cytokines, and different morphology^63–65^. Despite the limitations of the cell lines, the present work paves the way to a better understanding on the role of microglial A_2A_R as a putative target for the treatment of AMD. Nevertheless, more studies are needed, using primary cultures and animal models of AMD to ascertain the therapeutic potential of A_2A_R antagonists.

In summary, in previous studies, we have suggested that selective A_2A_R blockade might be seen as a possible therapeutic strategy for glaucoma^24–26,66^. In this work, using *in vitro* models, we show that an adequate modulation of microglial cell reactivity by blocking A_2A_R also presents beneficial effects on the modulation of the complement system, and might impact the microglia crosstalk with RPE and photoreceptors.

## Methods

### Cell lines and pharmacological compounds

The immortalized human microglia-SV40 cell line derived from primary human microglia was purchased from Applied Biological Materials, Inc. (ABM, Inc.). 661 W photoreceptor-like cells were a kind gift from Prof. Muayyad Al-Ubaidi (Department of Cell Biology, University of Oklahoma Health Sciences Center, Oklahoma City, OK, USA). ARPE-19 cells were obtained from ATCC (CRL-2302). Zymosan A from *Saccharomyces cerevisiae* and Phorbol 12-myristate 13-acetate (PMA) were purchased from Sigma-Aldrich. The A_2A_R antagonist SCH58261 was purchased from Merck Millipore.

### Cell cultures and treatments

**Figure 10:**
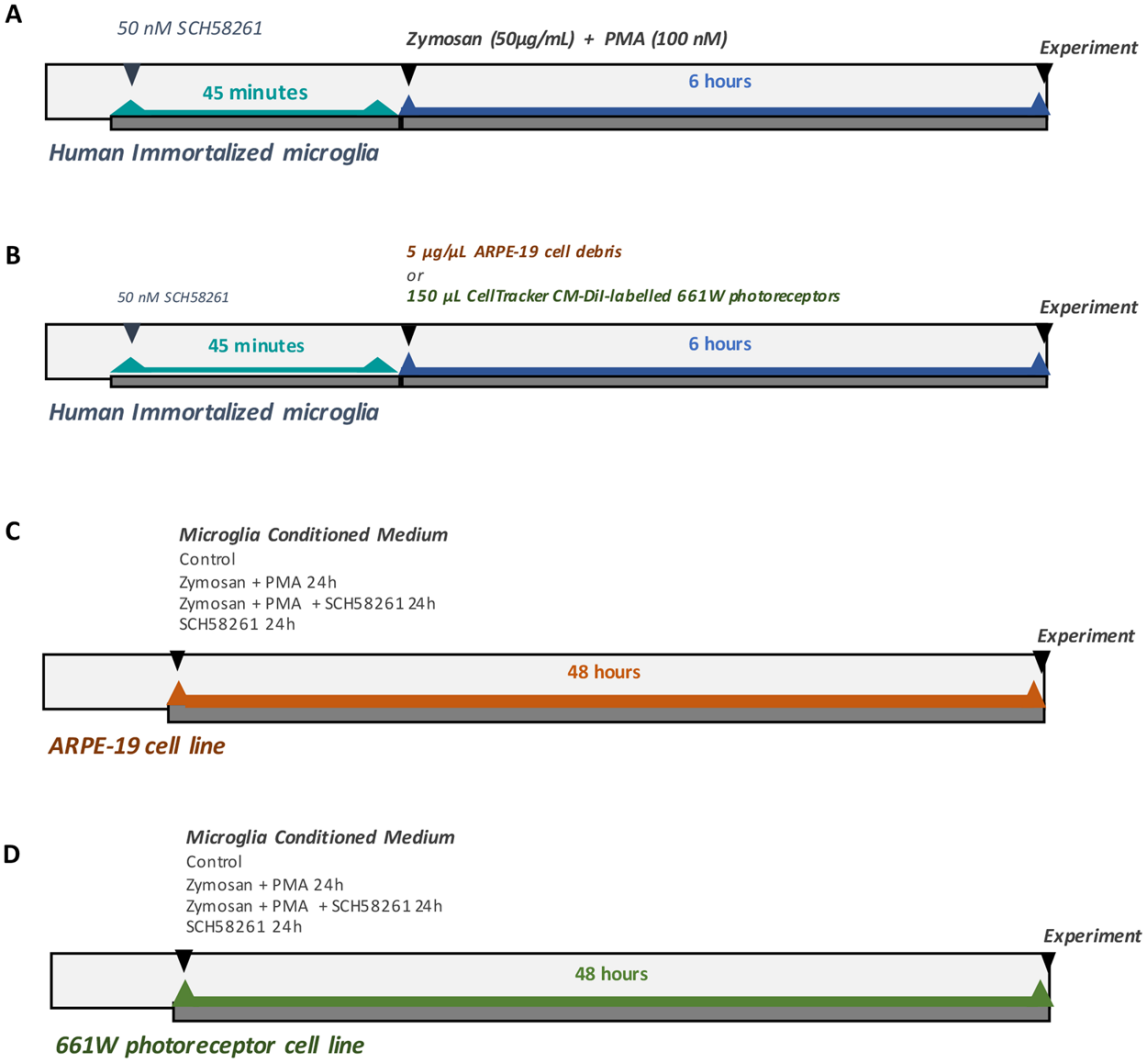
Schematic overview of the experimental design. (**A)** Human immortalized microglial cells were pretreated for 45 min with 50 nM SCH58261 (A_2A_R antagonist) and then challenged with Zymosan A (50 µg/mL) and PMA (100 nM) for 6 h. **(B)** Human immortalized microglial cells were challenged with 5 μg/μL ARPE-19 cell debris for 6 h, previously untreated or treated with 50 nM SCH58261. **(C)** ARPE-19 cell line was cultured with MCM for 48 h. **(D)** 661 W photoreceptor cell line was cultured with MCM for 48 h.

Human microglial cells were grown in collagen-coated T-75 cm^2^ flasks and cultured in high glucose Dulbecco’s modified Eagle’s medium (DMEM) supplemented with 10% fetal bovine serum (FBS) and 1% penicillin/streptomycin, at 37 °C in a humidified atmosphere of 5% CO_2_. Microglia were pretreated for 45 min with 50 nM SCH58261 (A_2A_R antagonist) and then challenged with Zymosan A (50 µg/mL) and PMA (100 nM) for 6 h. For immunocytochemistry and assessment of mRNA expression, microglial cells were plated at a density of 1 × 10^4^ cells and 1 × 10^5^ cells/cm^2^, respectively, in collagen-coated cover-slips on 12-well or 6-well plates (Fig. 10A).

Since drusen may result from cellular remnants and debris derived from degenerated RPE cells, debris obtained by serum-starved ARPE-19 cells were used to model the composition of drusen *in vitro*, as we reported previously^9^. To obtain RPE debris, ARPE‐19 cells were starved by serum deprivation for 2 weeks, harvested, centrifuged, and washed three times with PBS, and the pellet was frozen at −20 °C. Microglial cells were incubated with 5 μg/μL ARPE-19 cell debris for 6 h, previously treated or not with 50 nM SCH58261 (Fig. 10B).

ARPE-19 cell line was cultured in DMEM/F12 supplemented with 10% FBS and 1% penicillin/streptomycin. One day after seeding, the MCM was added to ARPE-19 cells for 48 h (Fig. 10C).

661 W photoreceptor cells were cultured in high glucose DMEM with L-glutamine, supplemented with 10% FBS and 1% penicillin/streptomycin, and maintained at 37 °C in a humidified atmosphere of 5% CO_2_. One day after seeding, the MCM was added to 661 W cells for 48 h (Fig. 10D).

### Adenosine quantification

The extracellular concentration of adenosine was quantified in human microglial cells supernatant using a fluorometric kit (BioVision), according to the instructions provided by the manufacturer.

### Immunocytochemistry and image analysis

Cells were fixed with 4% formaldehyde, washed with PBS, and incubated in blocking buffer containing 10% goat serum and 0.3% Triton X-100. Subsequently, cells were incubated with the antibodies rabbit anti-Iba1 (1:150; Wako) or rabbit anti-A_2A_R (1:200; Thermo Scientific) in a solution containing 2.5% goat serum and 0.1% Triton X-100 for 1 h at room temperature. Cells were incubated with goat anti-rabbit Alexa 488 (A-11012, Life Technologies) or with 0.1 μg/ml Phalloidin-TRITC (Sigma-Aldrich) (in the case of A_2A_R labeling). Cells were then washed with PBS and nuclei were stained with 4′,6-diamidino-2-phenylindole (DAPI). Cover slips were mounted with fluorescent mounting medium (Dako), and fluorescence photomicrographs were taken with an AxioImager.M2 microscope (Carl Zeiss).

Densitometric analysis of A_2A_R staining was performed using ImageJ software (National Institutes of Health, Bethesda, MD, USA) and mean grey value was calculated as previously described^25^, with the formula:

$${\rm{CTCF}}={\rm{Integrated}}\,\mathrm{density}-(\mathrm{area}\,{\rm{of}}\,{\rm{selected}}\,{\rm{cell}}\,\times \,{\rm{mean}}\,{\rm{fluorescence}}\,{\rm{of}}\,{\rm{background}}\,\mathrm{reading})$$

### Real-time quantitative Polymerase Chain Reaction (PCR)

Total RNA was extracted from human microglial cells or ARPE-19 using the NucleoSpin® RNA Mini Kit (Macherey-Nagel), according to the manufacturer’s instructions. RNA was quantified spectrophotometrically using a NanoDrop 2000 (Thermo Scientific) and then stored at −80 °C. Amplification of cDNA was with RevertAid™ H Minus First strand cDNA Synthesis Kit (Thermo Scientific), according to the instructions provided by the manufacturer, using 1 µg of total RNA.

Amplification of 50 ng cDNA was conducted in an ABI7900HT machine (Applied Biosystems) in 10 μl reaction mixtures containing 1 × TaqMan Universal PCR Master Mix (Applied Biosystems), 200 nM of primers, and 0.125 μL of dual-labeled UPL probe (Roche Applied Science). The reaction parameters were as follows: 2 min 50 °C hold, 30 min 60 °C hold, and 5 min 95 °C hold, followed by 45 cycles of 20 s 94 °C melt and 1 min 60 °C anneal/extension. Primer sequences and UPL probe numbers are listed on Table 1. Ct values were converted to “relative quantification” using the 2∆∆Ct method, using the method described by Livak^67^. *Hprt* was used as housekeeping gene.

**Table 1:** Primers used in qPCR and RT-PCR.

Gene	Primer Forward	Primer Reverse	Roche UPL probe numbers
Hprt	5′-tgaccttgatttattttgcatacc-3′	5′-cgagcaagacgttcagtcct-3′	73
Adora2A	5′-tgaccgctacattgccatc-3′	5′-tccaacctagcatgggagtc-3′	3
Ccl2	5′-agtctctgccgcccttct-3′	5′-gtgactggggcattgattg-3′	40
TNF	5′-cagcctcttctccttcctgat-3′	5′-gccagagggctgattagaga-3′	29
IL-8	5′-agacagcagagcacacaagc-3′	5′-cacagtgagatggttccttcc-3′	72
C3	5′-ccactggaggtgtctgacg-3′	5′-ctggtacaggcggatcttct-3′	1
CFB	5′-tccactgctatgacggttaca-3′	5′-gctgtctgcccactccat-3′	53
CD93	5′-ggccatggagaaccagtaca-3′	5′-ggaatggggagttcaaagc-3′	83
C5AR1	5′-ggagggaccttcgatcctc-3′	5′-ggggtggtataattgaaggagtt-3′	54
CFH	5′-gaatgggttgctcttaatcca-3′	5′-cctcctgtaagggtaaaagtacca-3′	80
CFI	5′-tggaccaagataagacaatgttca-3′	5′-accttctttgagtatcagcacctt-3′	78
CD46	5′-cggtaagcccccaatatgt-3′	5′-acttcactaaaggtgtgttttcca-3′	10
CD55	5′-ctgctggtgctgttgtgc-3′	5′-tcctcgggaaaacttgtacg-3′	20
C1s	5′-cttgcggagagaagattttga-3′	5′-ggaccaaattgccgatctc-3′	38
CASP-1	5′-ccaggacattaaaataaggaaactgta-3′	5′-ccaaaaacctttacagaaggatctc-3′	4
IL-18	5′-aacaaactatttgtcgcaggaat-3′	5′-tgccacaaagttgatgcaat-3′	46
IL-1β	5′-tacctgtcctgcgtgttgaa-3′	5′-tctttgggtaatttttgggatct-3′	78
NLRP3	5′-aaagagatgagccgaagtgg-3′	5′-atccactcctcttcaatgctg-3′	72

### Nitrite measurement

Nitric oxide concentration was estimated in the supernatant of human microglial cells by quantifying the concentration of nitrites using the Griess reaction method (Promega), as previously described and according to the manufacturer’s instructions. The results are presented as percentage of control.

### Microparticles phagocytosis assay

After platting and overnight seeding, microglial cells were treated as described above and 2 h after incubated with 2 µL of micro-particles based on polystyrene solutions (Sigma-Aldrich) for 6 h. After washing 3 times with PBS, 5 images were randomly acquired for each condition under an inverted microscope (Carl Zeiss). Phagocytic efficiency was calculated as described previously^24^. Results are presented as percentage of control.

### 661 W apoptotic material phagocytosis assay

661 W photoreceptor cells were starved by serum deprivation and harvested and fluorescently labeled using CellTracker CM-DiI (Invitrogen, Carlsbad, CA, USA). Human microglial phagocytic efficiency was assessed as previously described^59^. Briefly, microglial cells were treated with 150 µL of apoptotic 661W-labeled solution for 6 h in the presence or absence of SCH58261 (50 nM) (Fig. 10B). Cells were then fixed with 4% formaldehyde and immunolabeled with an antibody rabbit anti-Iba1 (1:150; Wako)^68^, as described above. Preparations were observed with an inverted fluorescence microscope AxioImager.M2 microscope (Carl Zeiss) and from each experimental condition, at least 5 fields were randomly acquired. Phagocytic efficiency was determined using the formula previously described^24^. Results are presented as percentage of control.

### 661 W photoreceptor apoptosis assay

Microglia-induced neurotoxicity was investigated as previously described^59^. Briefly, 661 W photoreceptor cells were incubated for 48 h with culture supernatants from control-, Zymosan + PMA-, Zymosan + PMA + SCH58261-, or SCH58261-treated microglial cells. Apoptotic cell death was determined using the Caspase-Glo® 3/7 Assay (Promega), according to the manufacturer’s instructions. Relative luciferase units (RLUs) reflect the level of apoptotic cell death. Results are presented as percentage of control.

### Statistical analysis

Normality of the data was assessed with Shapiro-Wilk normality test. Since data did not follow a Gaussian distribution, all data was analyzed using non-parametric tests: Mann-Whitney test (for comparison of two groups) or Kruskall-Wallis test (when comparing more than two groups), followed by Dunn’s multiple comparison correction test, as indicated in the figure legends. The values of P are presented above the bars for each comparison. The results are presented as mean ± standard error of the mean (SEM) and the individual data points are also shown. Values of P < 0.05 were considered statistically significant. The statistical analysis was performed in Prism 7.0 Software for Mac OS X (GraphPad Software, Inc).

## Electronic supplementary material

Supplementary Figures
