# Supplementary material for: Blockade of microglial adenosine A2A receptor impacts inflammatory mechanisms, reduces ARPE-19 cell dysfunction and prevents photoreceptor loss in vitro
Source: Sci Rep. 2018 Feb 2;8:2272. doi: 10.1038/s41598-018-20733-2 (PMC5797099; doi:10.1038/s41598-018-20733-2)
Supplement: Supplementary file 1 — Supplementary Figures [file 41598_2018_20733_MOESM1_ESM.pdf]

Supplementary Information

Blockade of microglial adenosine A2A receptor impacts inflammatory mechanisms, reduces ARPE-19 cell dysfunction and prevents photoreceptor loss in vitro

M.H. Madeira[1,2,3], K. Rashid[3], A.F. Ambrósio[1,2,4], A.R. Santiago[1,2,4], T. Langmann[3*]

[1]Institute for Biomedical Imaging and Life Sciences (IBILI), Faculty of Medicine, University of Coimbra, Portugal;

[2]CNC.IBILI Consortium, University of Coimbra, Portugal;

[3]Laboratory for Experimental Immunology of the Eye, Department of Ophthalmology, University of Cologne, Germany;

[4]Association for Innovation and Biomedical Research on Light and Image (AIBILI), Coimbra, Portugal.

*Corresponding author:

Professor Thomas Langmann

Email: thomas.langmann@uk-koeln.de

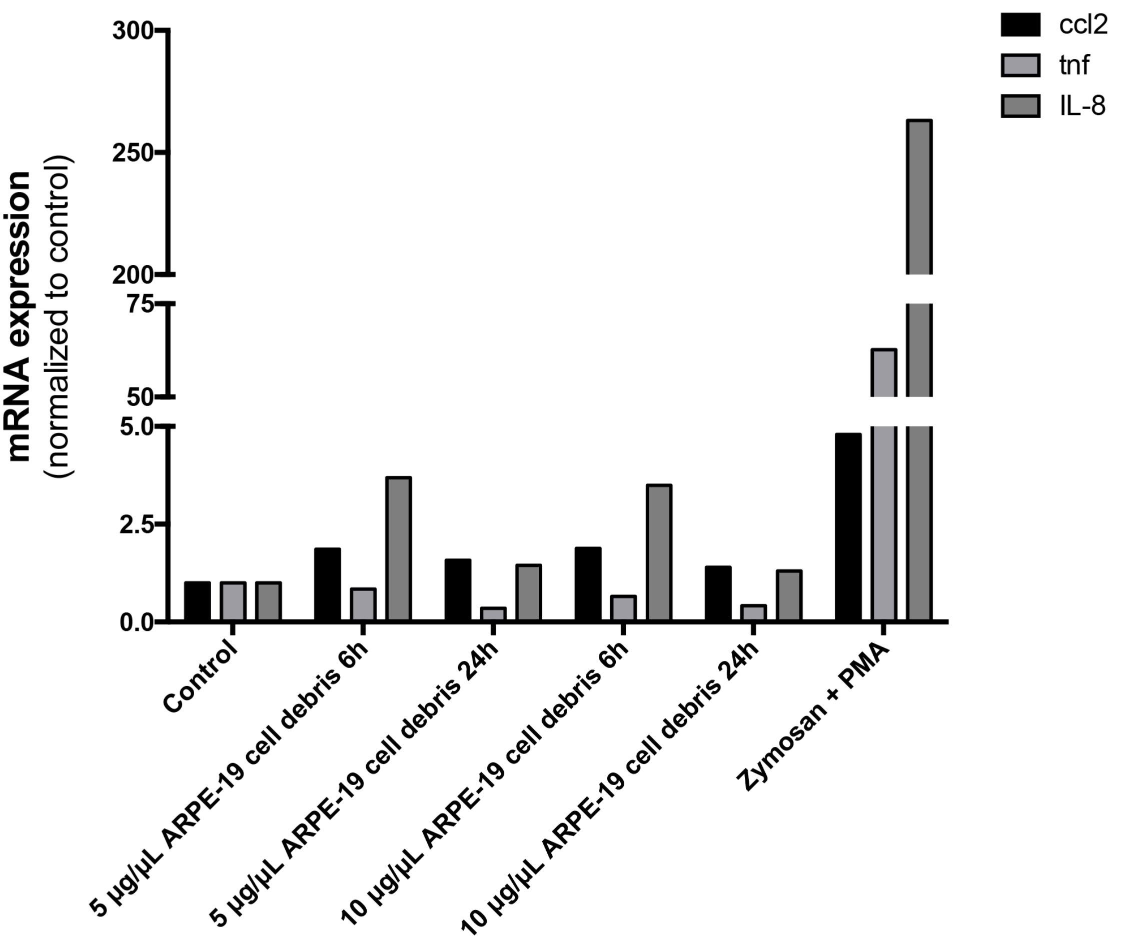

Supplementary figure 1 – Determination of the best time point and amount of ARPE-19 cell debris for microglia incubation.

Human microglial cells were treated with 5 or 10 µg/µL of ARPE-19 cell debris, for 6h or 24h, and the mRNA levels of CCL2**,** TNF and IL-8 were assessed by qPCR. Results are normalized to control and presented as the mean ± SEM of 3 independent experiments. Treatment with Zymosan+PMA for 6h was used as a positive control.

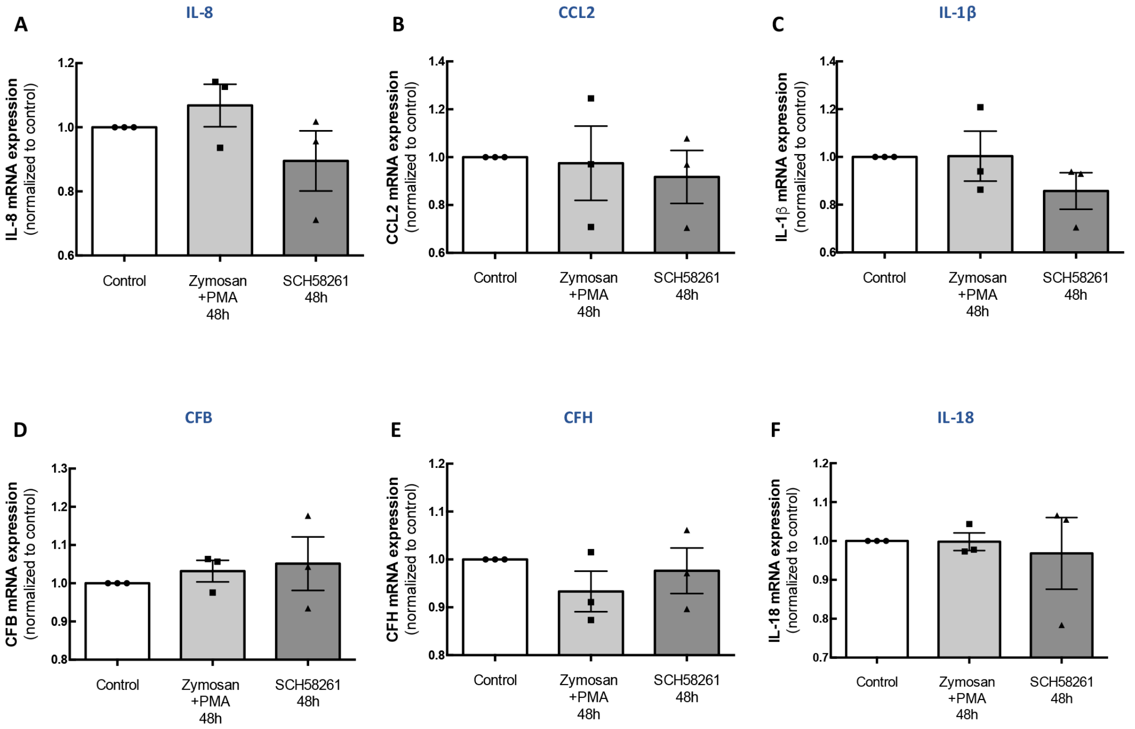

Supplementary figure 2 – Effect of Zymosan+PMA treatment on the expression of inflammatory and complement system components in ARPE-19 cells.

ARPE-19 cells were incubated with Zymosan+PMA for 48 h and the mRNA expression of IL-8 **(A)** CCL2 **(B),** IL-1β **(C)**, CFB **(D)**, CFH **(E)** and IL-18 **(F)** was assessed by qPCR. Results are normalized to control and represent the mean ± SEM of 3 independent experiments (presented with the scatterplot with the individual data points).

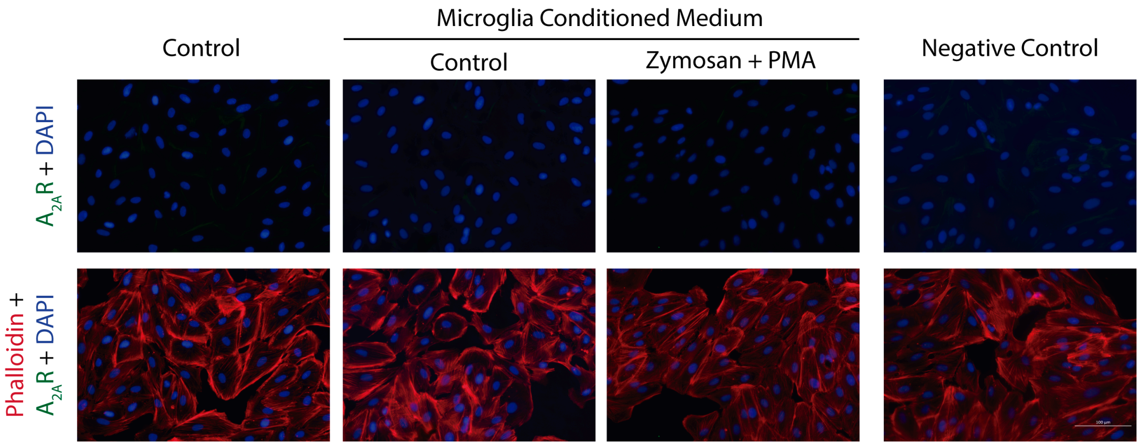

Supplementary figure 3 – ARPE-19 cells lack $A_{2A}R$ immunoreactivity.

ARPE-19 cells were immunostained for $A_{2A}R$ (green) and phalloidin (red). Nuclei were counterstained with DAPI (blue). Representative images obtained from 3 independent experiments.

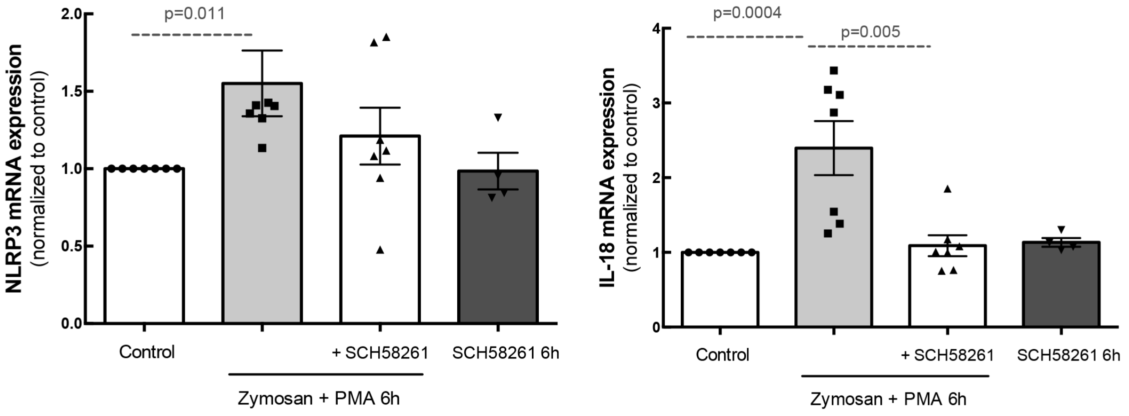

Supplementary figure 4 – Blockade of $A_{2A}R$ prevents the alterations in inflammasome in reactive human microglial cells.

Human microglial cells were challenged with Zymosan + PMA in the absence or presence of 50 nM SCH58261. The expression levels of complement cascade components NLRP3 **(A)** and IL-18 **(B)** were assessed by qPCR. Results are normalized to control and presented as the mean ± SEM of 7 independent experiments (the individual data points for each condition are also presented). Kruskall-Wallis test, followed by Dunn's multiple comparison test.
